# Reply to Lei et al. Comment on “Altobelli et al. Colon Capsule Endoscopy as a Promising Diagnostic Tool in Colorectal Cancer: A Systematic Review and Network Meta-Analysis. *Diagnostics* 2025, *15*, 2157”

**DOI:** 10.3390/diagnostics15232939

**Published:** 2025-11-21

**Authors:** Emma Altobelli, Paolo Matteo Angeletti, Francesco Masedu

**Affiliations:** 1 Section of Epidemiology and Public Health Unit, Department of Life, Health and Environmental Sciences, University of L’Aquila, Piazzale Salvatore Tommasi 1, Coppito, 67100 L’Aquila, Italy; 2 Cardiovascular Department, UO of Cardiac Anesthesia of the IRCCS Humanitas Research Hospital, Via Alessandro Manzoni 56, Rozzano, 20089 Milan, Italy; 3 Department of Applied Clinical Sciences and Biotechnology, University of L’Aquila, Via Vetoio, Coppito, 67100 L’Aquila, Italy; francesco.masedu@univaq.it

We sincerely thank the reviewer for their thoughtful and constructive comments on our network meta-analysis (NMA) evaluating colon capsule endoscopy (CCE) for colorectal cancer (CRC) screening [1,2]. We appreciate the opportunity to clarify our rationale, address methodological considerations, and situate our findings within the changing body of evidence.

## 1. Rationale for NMA Despite Existing Head-to-Head Studies

While multiple direct comparisons between CCE and colonoscopy (COL) exist, they are often limited in scope, vary in study populations, or do not include indirect comparisons with other relevant modalities. Our rationale for conducting an NMA was to synthesise both direct and indirect evidence across a broader network of diagnostic strategies to provide a more comprehensive and comparative assessment of CCE’s performance. This approach is particularly useful in identifying relative performance in contexts where direct evidence may be sparse or heterogeneous [3].

## 2. Use of Standardised Mean Differences (SMDs)

We acknowledge that the use of SMDs in diagnostic test accuracy (DTA) meta-analyses is unconventional. Formulas for transforming proportions in ORs have been proposed like SMD ≈ ln(OR)/1.81 (Chinn, S. (2000) [4]). We agree that our meta-analytical model is not optimal, as standardised mean differences (SMDs) are not designed for correlated diagnostic parameters such as sensitivity (Se) and specificity (Sp), which are mathematically and clinically linked changes in that one affects the other. Replacing SMDs with other effect measures, such as odds ratios or risk ratios, would not resolve this issue, since these parameters remain inherently correlated through the same contingency table. Analysing them separately, regardless of the effect size used, fails to capture their joint distribution.

We appreciate the suggestion regarding the Bayesian framework. Properly accounting for this correlation requires joint modelling approaches, such as bivariate or hierarchical summary ROC (HSROC) models (Rutter CM, Gatsonis CA. (2001) [5], often implemented within a Bayesian structure. Such models reduce bias arising from separate analyses of sensitivity and specificity and provide more coherent estimates of relative diagnostic performance.

## 3. Heterogeneity and the Role of Meta-Regression

We concur that heterogeneity, particularly due to differences in bowel preparation protocols and completion rates, may influence pooled estimates. Meta-regression was considered; however, the available data on relevant covariates were inconsistently reported across studies [5,6].

## 4. Remarks About the Distinctions Between CCE-1 vs. CCE-2

We agree that distinguishing between CCE generations is critical, and we appreciate the reference to Lei et al. (2025) [7]. As noted in our limitations, our pooled estimates may underrepresent the performance of second-generation CCE (CCE-2). Future studies will prioritise disaggregation by generation where feasible to provide a clearer picture of current capabilities.

## 5. Pragmatic Considerations and Real-World Implementation

We acknowledge the findings of Baatrup et al. (2025) [8], which highlight important real-world implementation challenges. As we noted in our discussion, while diagnostic performance is encouraging, broader integration of CCE into screening pathways must consider uptake, patient adherence, and downstream resource utilisation. Cost-effectiveness and workflow integration, especially when CCE is positioned as a second-line or alternative tool, require careful evaluation.
